# A Comparative Study of Cathepsin D Expression in Peripheral and Central Giant Cell Granuloma of the Jaws by Immunohistochemistry Technique

**Published:** 2016-06

**Authors:** Massoumeh Zargaran, Abbas Moghimbeigi, Noushin Afsharmoghadam, Mohsen Nasr Isfahani, Atefeh Hashemi

**Affiliations:** 1 Dental Research Center, Dept. of Oral and Maxillofacial Pathology, Dental School, Hamadan University of Medical Sciences, Hamadan, Iran.; 2 Modeling of Noncommunicable Disease Research Center, Dept. of Biostatistics and Epidemiology, School of Public Health, Hamadan University of Medical Sciences, Hamadan, Iran.; 3 Dept. of Pathology, AL Zahra Medical Center, Isfahan University of Medical Sciences, Isfahan, Iran.; 4 Pathology Technologist, Dept. of Pathology, AL Zahra Medical Center, Isfahan University of Medical Sciences, Isfahan, Iran.; 5 Dept. of Oral and Maxillofacial Pathology, Dental School, Arak University of Medical Sciences, Arak, Iran.

**Keywords:** Giant Cell Granuloma, Cathepsin D, Immunohistochemistry, Jaw

## Abstract

**Statement of the Problem:**

Peripheral and central giant cell granuloma are two common benign lesions of the oral cavity. In spite of histopathological similarities, they have different clinical behaviors. Cathepsin D is a lysosomal enzyme which has different functions on the basis of protein and applied peptide cleavage.

**Purpose:**

This research aimed to evaluate and compare the expression level of Cathepsin D in these two lesions to find the reasons for the differences in clinical and biologic characteristics.

**Materials and Method:**

The expression of Cathepsin D was investigated by using the immunohistochemistry method in 20 samples of peripheral giant cell granuloma and 20 samples of central giant cell granuloma. The percentage of stained giant cells (labeling index), the intensity of staining of giant cells, and staining-intensity-distribution in both groups were calculated and compared.

**Results:**

The labeling indices of Cathepsin D in peripheral giant cell granuloma and central giant cell granuloma were 95.9±4.03 and 95.6±2.34, respectively. There was no significant difference in the percentages of stained giant cells between the two groups (*p*= 0.586). The intensity of staining of giant cells in central giant cell granuloma was stronger than that of peripheral giant cell granuloma (*p*> 0.001). Staining- intensity- distribution of giant cells in central giant cell granuloma was significantly greater than that of the peripheral type of lesion (*p*= 0.001).

**Conclusion:**

The higher expression level of Cathepsin D in central giant cell granuloma compared to peripheral type of lesion can explain more aggressive behavior of central giant cell granuloma.

## Introduction

Peripheral giant cell granuloma (PGCG) occurs as a red or purple nodule exclusively on the gingiva and alveolar ridge. These lesions originate from the periodontal ligament or mucoperiosteum of the alveolar ridge as a result of local irritation or trauma.[1] PGCG can develop at any age, especially during the fifth and sixth decades of life with a slight female predilection.[1-2]

In some cases, PGCG affects the underlying bone and may cause cupping resorption.[2] Central giant cell granuloma (CGCG) occurs within the jaw bones and appears as radiolucent defects in radiographs which may be unilocular or multilocular.[3] The majority of these lesions are noted in young adults with a predilection for females.[4-5] Even some case of this lesion have been associated with significant asymmetries,[6-7] repeated recurrences,[8] multifocal incidences,[8-9] invasion and extensive destruction of jaw bones.[10] Both CGCG and PGCG exhibit similar histopathological features, and are characterized by the presence of abundant mononuclear stromal cells, admixed with a large number of multinucleated giant cells and a rich vascularized stroma with extravasated erythrocytes, hemosiderin deposition, and blood. Meanwhile, these lesions may have different clinical behaviors.[11-12]

Although various parameters have been compared between these two lesions in different studies, the reasons behind the differences in the biologic behaviors of these lesions are still to be elucidated.[3, 13-14] Cathepsin D is a soluble lysosomal aspartic endopeptidase which is released from the rough endoplasmic reticulum as preprocathepsin D and after elimination of the signal peptide, procathepsin D is carried into the intracellular vesicular structures. The various physiologic functions of Cathepsin D depend on its capacity to cleave functional and structural proteins and peptides.[15] Research has shown the presence of Cathepsin D in the vacuoles and vesicles of alveolar bone osteoclasts in mice, indicating that they are necessary for bone resorption and remodeling.[16-17] In addition, it has been demonstrated that giant cells in the giant cell tumor of long bones contain Cathepsin D. This enzyme has a positive relationship with the local invasion of this tumor, which might be attributed to its role in bone resorption.[18] Given the presence of Cathepsin D in osteoclasts and its effect on resorption of bone, the present study was undertaken to evaluate and compare its incidence through an immunohistochemical technique in PGCG and CGCG in an attempt to explain the reasons for different biological behaviors of these two tumors.

## Materials and Method

The present descriptive analytical study was carried out by using a cross-sectional design. A total of 40 samples were selected from the archives of the Department of Oral Pathology, Faculty of Dentistry of Hamadan University of Medical Sciences and the archives of the department of pathology, AL Zahra Medical Center, Isfahan University of medical sciences in two groups. Group A consisted of 20 samples of PGCG and group B consisted of 20 samples of CGCG. The CGCG samples were selected based on the occurrence of central lesions in the jaws as shown on radiographic views (if available) or according to the report made by the radiologist in the patient’s medical file (if radiograph was not available). Cases without sufficient documents were excluded from the study. An indirect immunohistochemistry technique was used to evaluate and compare the samples.

**Immunohistochemical Technique**

The indirect immunohistochemical technique to stain for Cathepsin D consisted of the following steps:

1. Deparaffinization step: the slides with the specific tissue were placed in an oven for 60 minutes and heated to 60° C. They were then immersed in xylol and descending concentrations of alcohol (90%, 80% and 70%, respectively) for the tissue water absorption in preparation for the test.
2. Hydrogen peroxide step: The prepared slides were placed in 3% hydrogen peroxide, and were rinsed in distilled water.
3. The Cathepsin D primary antibody step: Cathepsin D antibody (Rabbit Polyclonal Antibody, PU 205-UP, BioGenex, USA) was poured on the slides and rinsed with PBS buffer solution after one hour of incubation at room temperature.
4. Envision step: the secondary Envision antibody was applied on the slides for 30 minutes and then the slides were incubated at room temperature.
5. Chromogen step: The chromogen used was DAD (Diaminobenzidine), which was applied on the slides for 5-10 minutes, and the slides were rinsed with distilled water.
6. Background staining step: Hematoxylin was used to stain the background by applying it on the slides for 5 minutes. The slides were rinsed with distilled water. Finally the slides were mounted with enthelan glue which is suitable for visualization under a light microscope. Based on the manufacturer’s instructions, the breast carcinoma cells were used as the positive controls, in which the cancerous cells exhibit cytoplasmic staining for the Cathepsin D marker.

**Immunohistochemical evaluation**

The stained giant cell counts were determined by counting these cells in 8 fields on each slide under a light microscope (Olympus B 40X; Japan) at 400 X magnification. Then the labeling index (LI) for Cathepsin D was calculated for each sample based on the number of stained cells. Counting procedures were carried out twice to avoid errors. The Cathepsin D LI (the percentage of stained giant cells) was analyzed and compared between group A (PGCG) and group B (CGCG).

In addition, the Cathepsin D immunoreactivity was evaluated semi-quantitatively and scored based on the percentage of stained cells ; score 0 when <1% of cells were stained, and score 1 to 4 when 1-25%, 26-50%, 51-75%, and >75% of cells were stained respectively. 

Staining intensity was evaluated and scored as 0 (no cell staining), 1 (weak staining), 2 (moderate staining), and 3 (intense staining) based on the stating strength of the cells.[19]

The scores were determined in each field. In order to achieve more accurate results and more reliable determination of staining intensity in the whole slide, 8 fields were scored separately and their means were calculated. 

Staining intensity distribution (SID) was calculated separately for each field by multiplying the percentage of giant cells stained in that field by the intensity of staining in that field; the mean SID was calculated for each sample. 

**Statistical Analyses**

Statistical analyses were carried out by using SPSS software, version 16. Kolmogorov-Smirnov was used to determine the distribution of the three variables of stained cells, staining intensity, and SID. 

T test was used to evaluate Cathepsin D LI and SID between the two groups. Fisher’s exact test was used to evaluate and compare the staining intensity of giant cells between the two groups. 

## Results

By assessment of study group documents, the patients’ information such as age, sex, and site of the lesions were extracted. (tables 1 and 2)

**Table 1:** Frequency distribution of the two study groups regarding gender and age

**Group**	**No.**	**Gender**		**Age**			
		**Male**	**Female**	**Mean**	**SD**	**Maximum**	**Minimum**
A	20	(45%)9	(55%)11	31.6	20.34	65	7
B	20	(40%)8	(60%)12	32.75	16.48	66	8
Total	40	(42.5%)17	(57.5%)23				

**Table 2:** Frequency distribution of the two study groups regarding the site of lesions

**Group**	**N**	**Site of the samples**					
		**Anterior jaw ** **(unilateral)**	**Anterior jaw** **(bilateral)**	**Posterior jaw**	**Anterior and ** **Posterior jaw**	**Unknown**	**Total**
A	20	10 (50%)	1 (5%)	5 (25%)	4 (20%)	0 (0%)	20 (100%)
B	20	8 (40%)	1 (5%)	6 (30%)	4 (20%)	1 (5%)	20 (100%)

**Immunohistochemical findings**

**Figure 1:**
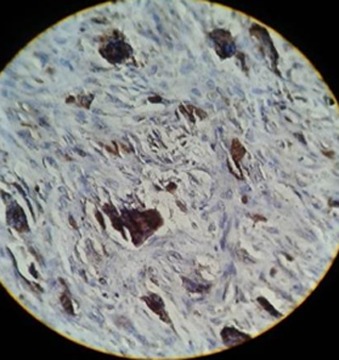
Staining of giant cells for Cathepsin D in CGCG

**Figure 2:**
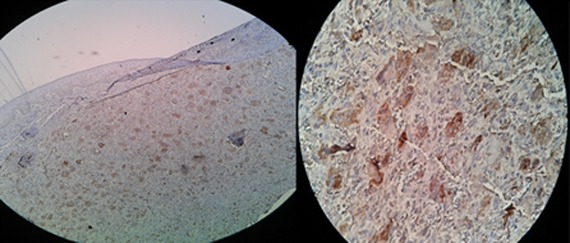
Staining of giant cells for Cathepsin D in PGCG

Positive immunostaining giant cells had cytoplasmic brown staining in the two groups (figures 1 and 2).

Kolmogorov-Smirnov test showed normal distribution of the three variables of stained giant cells, staining intensity, and SID. The mean percentage of stained giant cells was 95.09±4.03 and 95.6±2.34 in groups A and B respectively, with no statistically significant differences between the two groups (*p*= 0.586).

Table 3 presents the results of staining intensity of giant cells in the two study groups. The results of statistical analyses showed significant differences in staining intensity between the two groups. The mean SID in groups A and B was 7.140±1.40 and 9.21±1.90, respectively, indicating statistically significant differences between the two groups.

**Table 3:** The staining intensities for Cathepsin D in the two study groups

**Group**	**Staining Intensity**				
	**Score 0 (no staining)**	**Score 1 (weak staining)**	**Score 2 (moderate staining)**	**Score 3 (severe staining)**	
A	0 (0%)	13 (65%)	7 (35%)	0 (0%)	20 (100%)
B	0 (0%)	4 (20%)	14 (70%)	2 (10%)	20 (100%)
Total	0 (0%)	17 (45%)	21 (52.5%)	2 (5%)	40 (10%)

## Discussion

In the present study, expression of Cathepsin D was detected in the giant cells of both CGCG and PGCG lesions. Different studies evaluated the giant cells in these two lesions and all showed the osteoclastic nature of these cells.[19-20]

On the other hand, Goto *et al.* evaluated the growth plates of the femur and detected large amounts Cathepsin D suggesting that this enzyme is necessary for osteoclastic resorption.[16] In another study, Goto *et al.* showed that Cathepsin D has an active indirect role in osteoclastic resorption.[17] Demertzis *et al.* carried out a study on giant cell tumor and reported that all the giant cells in all samples expressed this enzyme. They reported a significant relationship between the expression of Cathepsin D and local invasion and expansion of the tumor.[18] Therefore, the expression of Cathepsin D in giant cells, which is considered a factor involved in bone destruction and one of the enzymes found in osteoclasts, might confirm the osteoclastic nature of giant cells in both PGCG and CGCG.

Osteoclasts contain several enzymes for resorption of bone, including the Cathepsin group (A, B, Z, *etc.*). Czupalla *et al.* reported that Cathepsin D is a type of Cathepsin synthesized in osteoclasts.[21] As discussed above, various studies showed the osteoclastic nature of giant cells in PGCG and CGCG lesions. Thus, the presence and expression of this enzyme in these cells can be expected. However, the question is what the reason is behind the differences in the expression intensity of this enzyme in giant cells in these two lesions. Among the most important factors involved in the osteoclastogenesis and bone resorption are proinflammatory mediators. For example it was reported that TNF-α and IL-1 play an important role in the formation and activation of osteoclasts.[22] TNF-α exerts its osteoclastogenic effect in association with other cytokines such as IL-1β. In this context, mice that had no IL-1β, but received TNF-α, exhibited no bone destruction despite persistent inflammation.[23]

A study by Papanicolaou *et al.* found that TNF-α and IL-1β proinflammatory factors were expressed in both PGCG and CGCG lesions. However, IL-1β was expressed in CGCG giant cells at a significantly higher rate than that in PGCG giant cells.[24] As previously mentioned, TNF-α plays a role in osteolysis only in the presence of IL-1β. Since IL-1β is expressed in CGCG giant cells at a higher rate, TNF-α can be more effective in osteoclastogenesis of these cells, and consequently in production of its products such as Cathepsin D.[25]During osteoclastogenesis, the enzymes required for the activity of these cells (including Cathepsin D) are synthesized. Due to the apparent osteoclastic nature of giant cells in PGCG and CGCG, the more intense expression of Cathepsin D in CGCG might be attributed to more significant and noticeable presence of IL-1β cytokine in these cells.

Cathepsin D plays a role in various physiologic and pathologic processes including bone resorption.[16-18] A higher rate of its expression by CGCG giant cells compared to PGCG might help explaining the processes of bone invasion and more bone destruction in CGCG since Cathepsin D can play a role in the destruction of extracellular matrix (ECM) by affecting the proteins of ECM, proteoglycans, and collagen.[26-27] Therefore, a higher rate of its expression in CGCG lesions compared to PGCG might explain greater destruction in these lesions.

Likewise, Cathepsin D in osteoclasts plays an indirect role in the destruction of bone matrix through activation of Cathepsin B and L.[17] Therefore, a higher concentration of Cathepsin D in CGCG giant cells might be considered as a factor to produce more Cathepsin B and L that are active, and consequently more bone destruction. Cathepsin B and L that are active will prevent further osteolytic activity in the lesion.

Moreover, Moles *et al.* showed that under *in vitro* conditions, an increase in the expression of Cathepsin D resulted in an increase in the synthesis of TGF-β in hepatic cells.[28] Therefore, a higher rate of expression of Cathepsin D in CGCG compared to PGCG might lead to higher expression of TGF-β. The expression of TGF-β in CGCG lesions was higher than that in PGCG lesions. It was consistent with the results of a study reported by de Matos *et al.*[29] On the other hand; Quan *et al.* reported that TGF-β had an effective role in the bone resorption process which caused higher survival rate of osteoclasts and increased the capacity of MMP-9 in bone resorption.[30]

Zhang *et al.* showed that Src factor was activated by TGF-β in lung cancerous cells[31] and as mentioned above, de Matos *et al. *showed that TGF-β was expressed at a lower rate in PGCG compared to CGCG.[29] Khiavi *et al.* noticed that Src factor was expressed in both lesions, but at a higher rate in CGCG compared to PGCG.[19] Based on the study by Moles *et al.*, the increase in the expression of TGF-β was affected by Cathepsin D.[28] Therefore, its higher expression of Cathepsin D and TGF-β might explain a higher expression of Src in CGCG giant cells. Apart from its role in osteoclastogenesis, Src plays a significant role in the polarity of osteoclasts, formation of a brush border, and bone resorption.[32]

Hu *et al.* reported that Cathepsin D affected the activity of MMP-9 in cells; therefore, use of Pepstatin A, the inhibitor of Cathepsin D, *in vitro* (similar to the specific inhibitor of MMP-9) resulted in inhibition of this enzyme.[33] MMP-9 has a role in absorption of bone through proteolysis of the organic matrix of bone.[34] Matos *et al.* carried out a study on PGCG and CGCG and observed that MMP-9 was expressed in CGCG giant cells at higher rates compared to the PGCG giant cell.[35] Based on the results of the present study, a higher rate of expression of Cathepsin D in CGCG giant cells might be associated with higher expression of MMP-9 in these cells, resulting in greater bone destruction in these lesions. Rundhaug has also shown that a higher rate of expression of MMP-9 results in an increase in VEGF levels.[36] VEGF has a role in osteoclastogenesis as a recruiting agent for osteoclast progenitors[37] and a mediator for their differentiation, directly affect the differentiated osteoclasts, in addition to its role in angiogenesis.[34] Likewise, in the study by Matos, VEGF was expressed in CGCG giant cells at a higher rate compared to PGCG giant cells.[35] Therefore, Cathepsin D can also indirectly have a role in the resorption of bone through these mechanisms.

In an *in vitro* study, Diment *et al.* showed that Cathepsin D could convert parathyroid hormone (PTH) to its active form, PTHrP.[1-34,38] Constant exposure to PTH or PTHrP activates resorption of bone by osteoclasts. In addition, their effect halts the osteoblast maturation process.[39] Houpis *et al.* evaluated PGCG and CGCG samples and reported that PTHrP and its receptor (PTHR) were expressed in all samples at a higher rate in CGCG giant cells compared to PGCG giant cells.[40] Therefore, it might be suggested that a higher rate of expression of Cathepsin D in CGCG giant cells results in the synthesis of more PTHrP (the active form of PTH). Accordingly, Cathepsin in the presence of a higher concentration of PTHrP in CGCG giant cells might explain a higher rate of bone resorption in this lesion.

Based on the available data and a literature review, this study appears to be the first in which Cathepsin D was evaluated in PGCG and CGCG quantitatively and semi-quantitatively. Hence, the mechanisms suggested in the present study to explain a higher rate of expression of Cathepsin D in CGCG compared to PGCG should be further evaluated and confirmed by further studies. In addition, given the complexities and the extent of involved cellular-molecular mechanisms, the effect of these currently-unknown factors cannot be ruled out.

## Conclusion

The number of stained giant cells was similar in both groups (CGCG and PGCG) in the present study. The giant cells in CGCG stained more intensely than in PGCG. SID in the CGCG was significantly different from that in the PGCG group; it was higher in the former group. There was no relationship between the expression of Cathepsin D in the PGCG and CGCG giant cells and age and sex. 
